# Supplementary material for: Role of TLR4 signaling on Porphyromonas gingivalis LPS-induced cardiac dysfunction in mice
Source: PLoS One. 2022 Jun 1;17(6):e0258823. doi: 10.1371/journal.pone.0258823 (PMC9159598; doi:10.1371/journal.pone.0258823)
Supplement: S1 Data — (PDF) [file pone.0258823.s001.pdf]

Supplementary Figures of S1 Data

Role of TLR4 signaling on *Porphyromonas gingivalis* LPS-induced cardiac dysfunction in mice

Running title: Oxidative stress in the heart of periodontitis

Ichiro Matsuo [1,2], Naoya Kawamura [1,2], Yoshiki Ohnuki [1], Kenji Suita [1], Misao Ishikawa [3], Takehiro Matsubara [4], Yasumasa Mototani [1], Aiko Ito [5], Yoshio Hayakawa [1,6], Megumi Nariyama [7], Akinaka Morii [1,2], Kenichi Kiyomoto [1,2], Michinori Tsunoda [1,2], Kazuhiro Gomi [2], Satoshi Okumura [1]

[1] Department of Physiology, Tsurumi University School of Dental Medicine, Yokohama 230-8501, Japan

[2] Department of Periodontology, Tsurumi University School of Dental Medicine, Yokohama 230-8501, Japan

[3] Department of Oral Anatomy, Tsurumi University School of Dental Medicine, Yokohama 230-8501, Japan

[4] Division of BioBank, Center for Comprehensive Genomic Medicine, Okayama University Hospital, Okayama, Japan

[5] Department of Orthodontics, Tsurumi University School of Dental Medicine,

Yokohama 230-8501, Japan

[6] Department of Dental Anesthesiology, Tsurumi University School of Dental Medicine,

Yokohama 230-8501, Japan

[7] Department of Pediatric Dentistry, Tsurumi University School of Dental Medicine,

Yokohama 236-8501, Japan

*****Corresponding author: Satoshi Okumura:

Department of Physiology, Tsurumi University School of Dental Medicine,

2-1-3 Tsurumi, Tsurumi-ku, Yokohama 230-8501; (Tel. +81-(0)45-580-8476;

Fax. +81-(0)45-585-2889; e-mail: okumura-s@tsurumi-u.ac.jp)

Supplementary Figure 1

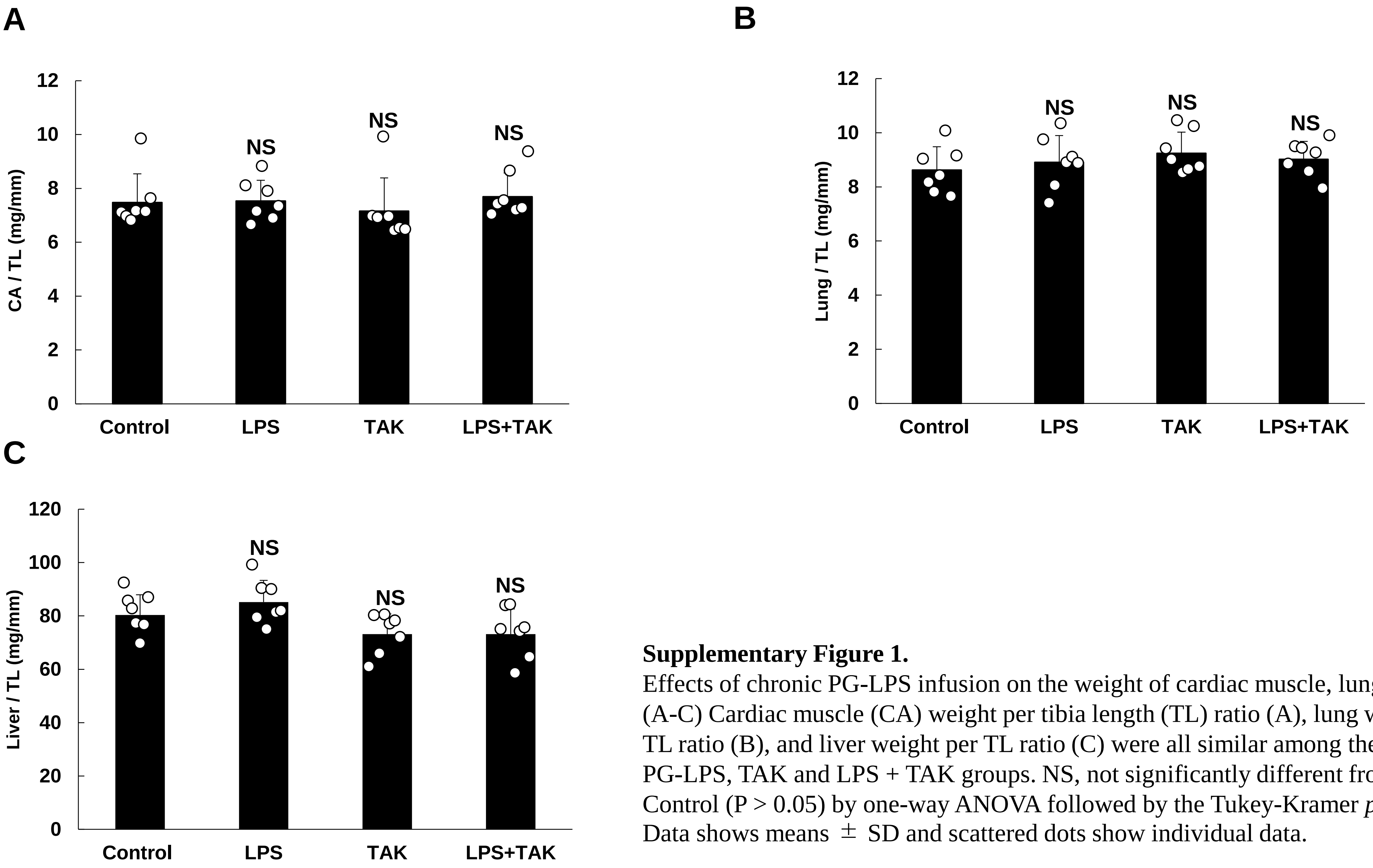

Supplementary Figure 1.
Effects of chronic PG-LPS infusion on the weight of cardiac muscle, lung and liver. (A-C) Cardiac muscle (CA) weight per tibia length (TL) ratio (A), lung weight per TL ratio (B), and liver weight per TL ratio (C) were all similar among the Control, PG-LPS, TAK and LPS + TAK groups. NS, not significantly different from the Control ($P > 0.05$) by one-way ANOVA followed by the Tukey-Kramer *post hoc* test. Data shows means ± SD and scattered dots show individual data.

Supplementary Figure 2

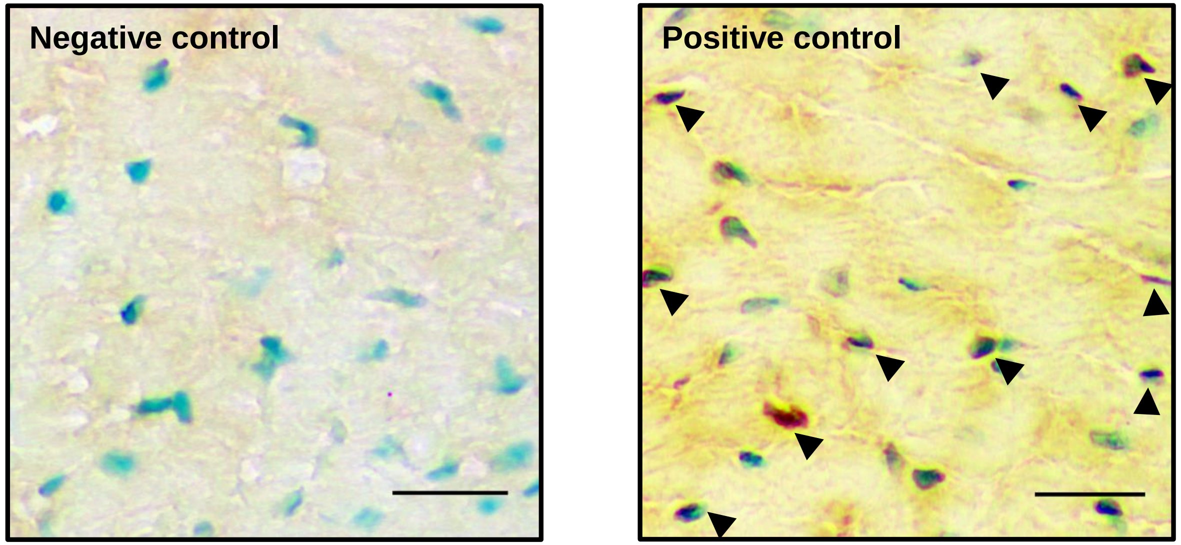

Supplemental Figure 2
Representative images of negative (left) and positive (right) controls of 8-OHdG immunostaining.
Scale bars: 2 μm

Supplementary Figure 3

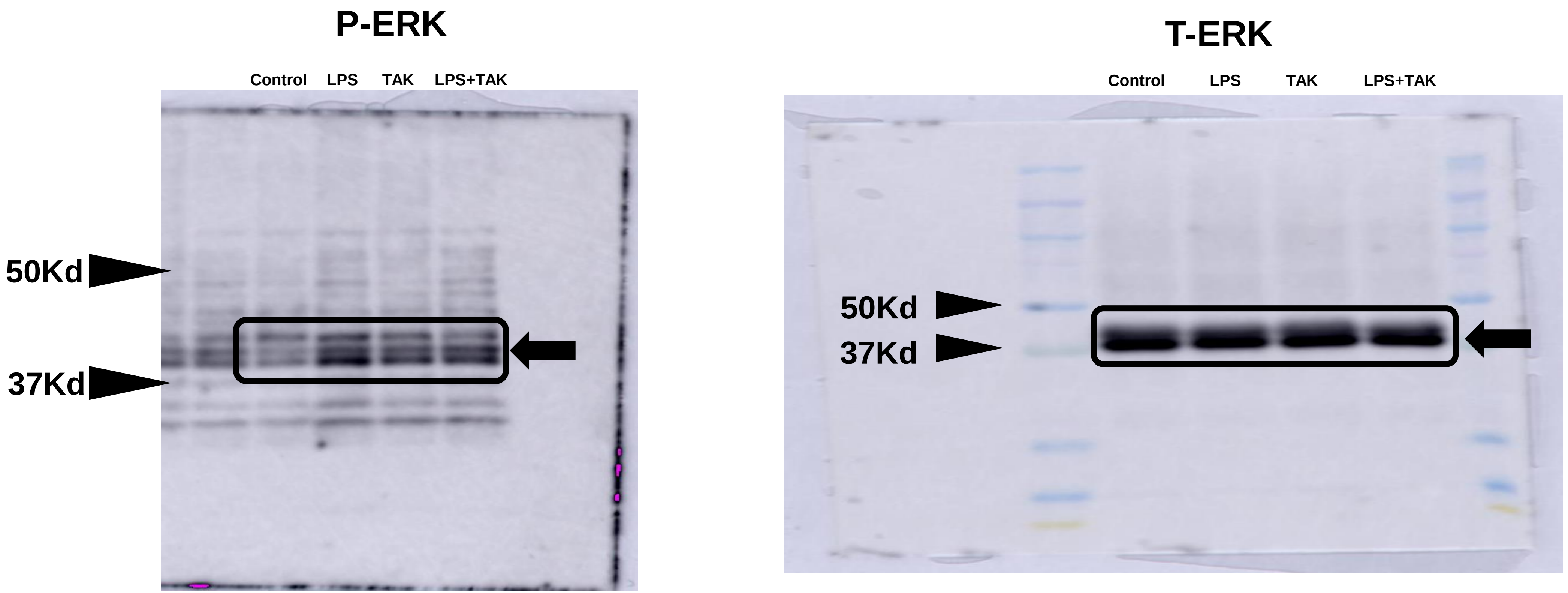

Supplementary Figure 3. Representative full-length immunoblots of Fig. 1C. The amount of P-ERK and T-ERK were shown. The black-line box indicated by arrow in each blot is corresponded to the cropped parts that are showed in the main article.

Supplementary Figure4

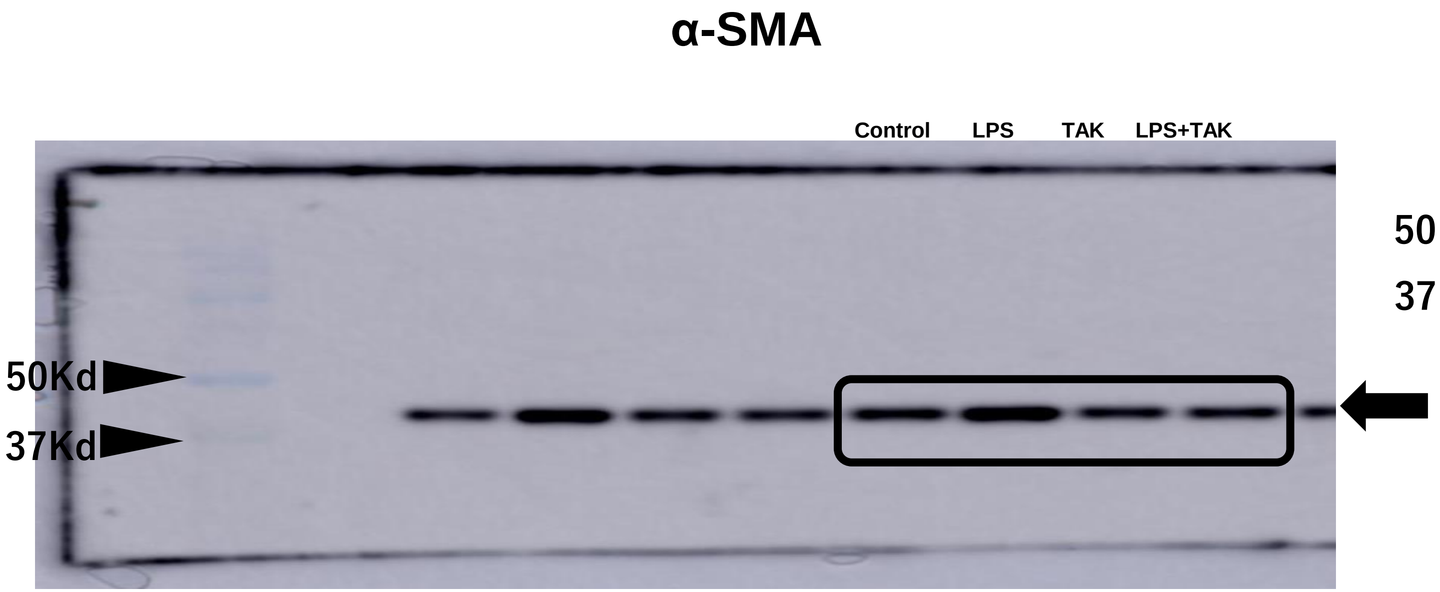

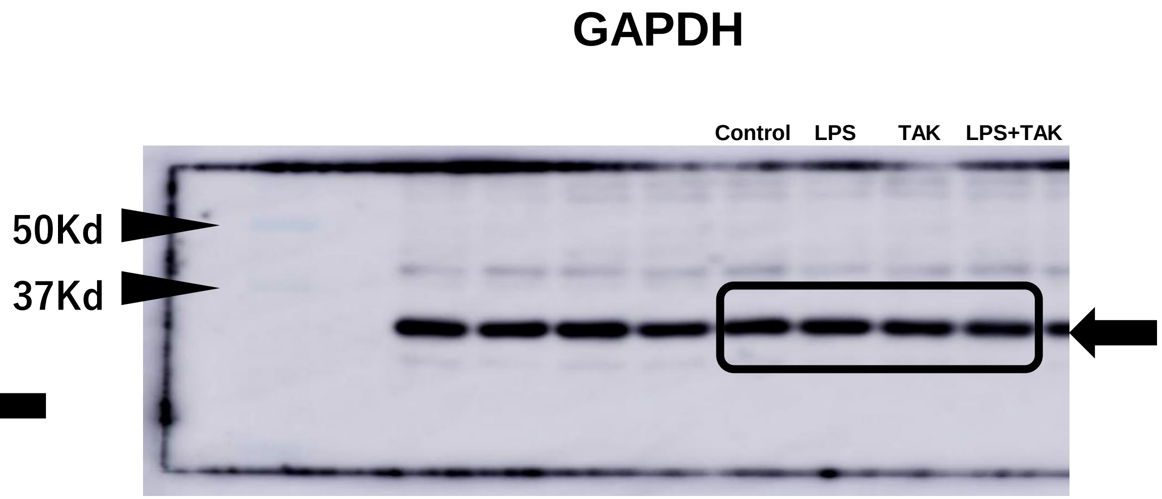

Supplementary Figure 4. Representative full-length immunoblots of Fig. 1D. The amount of α-SMA and GAPDH were shown. The black-line box indicated by arrow in each blot is corresponded to the cropped parts that are showed in the main article.

Supplementary Figure 5

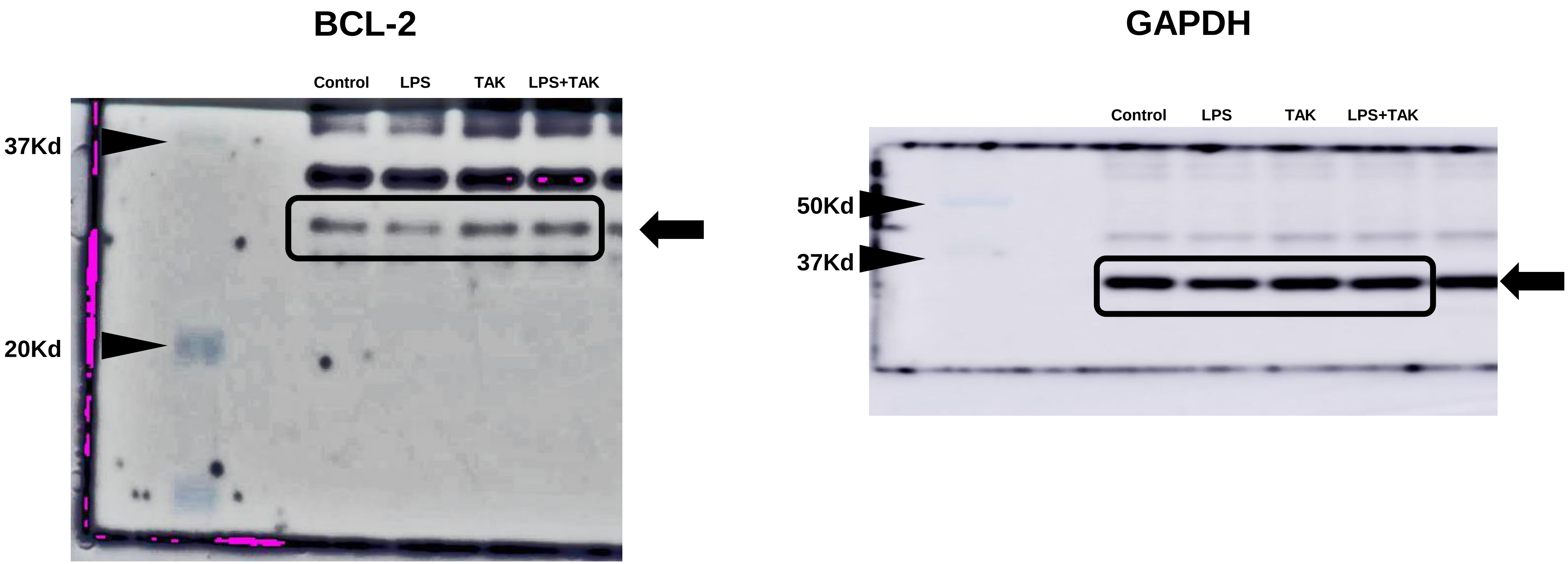

Supplementary Figure 5. Representative full-length immunoblots of Fig. 2C. The amount of BCL-2 and GAPDH were shown. The black-line box indicated by arrow in each blot is corresponded to the cropped parts that are showed in the main article.

Supplementary Figure 6

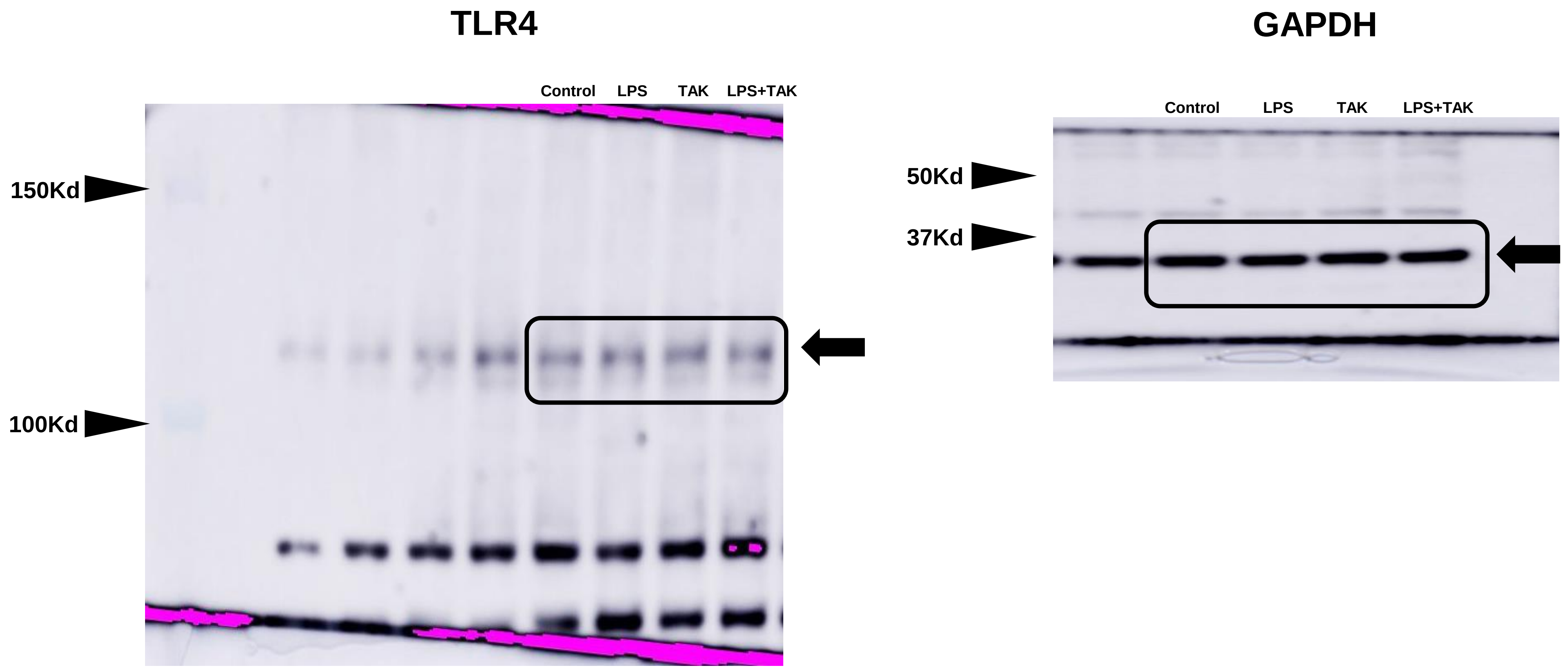

Supplementary Figure 6. Representative full-length immunoblots of Fig. 3C. The amount of TLR4 and GAPDH were shown. The black-line box indicated by arrow in each blot is corresponded to the cropped parts that are showed in the main article.

Supplementary Figure 7

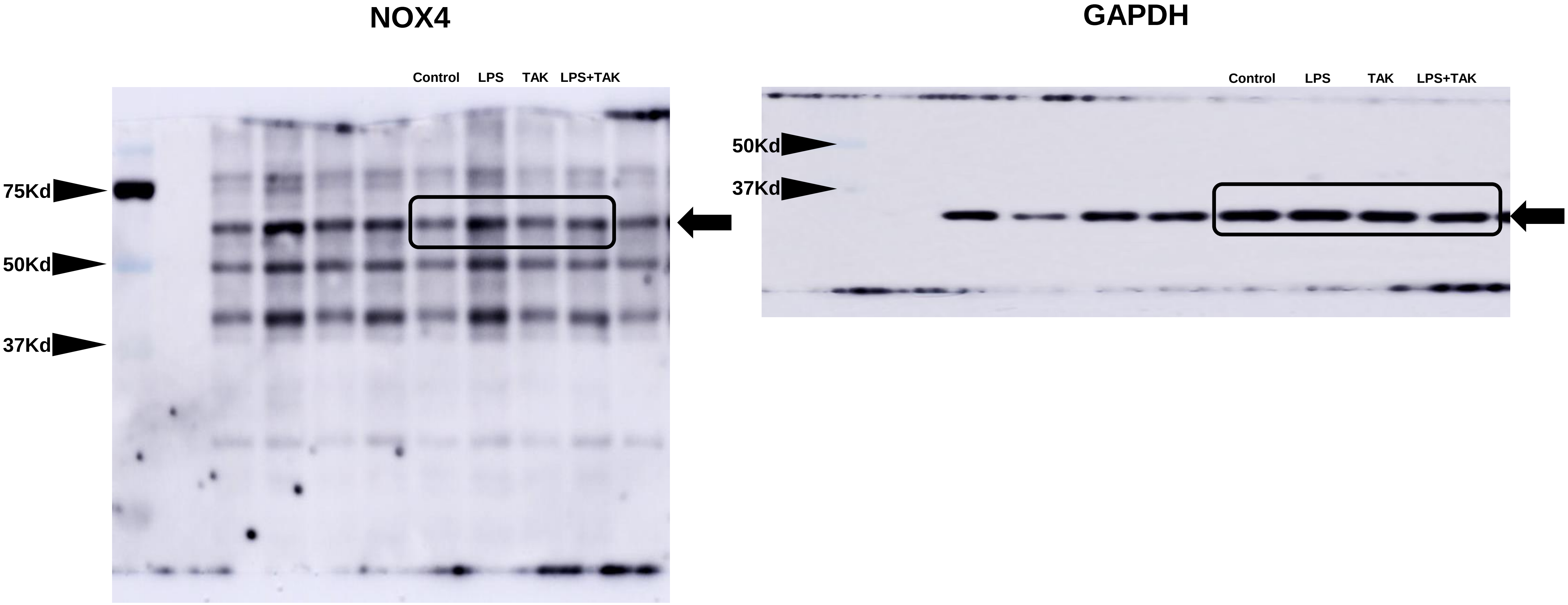

Supplementary Figure 7. Representative full-length immunoblots of Fig. 3D. The amount of NOX4 and GAPDH were shown. The black-line box indicated by arrow in each blot is corresponded to the cropped parts that are showed in the main article.

Supplementary Figure 8

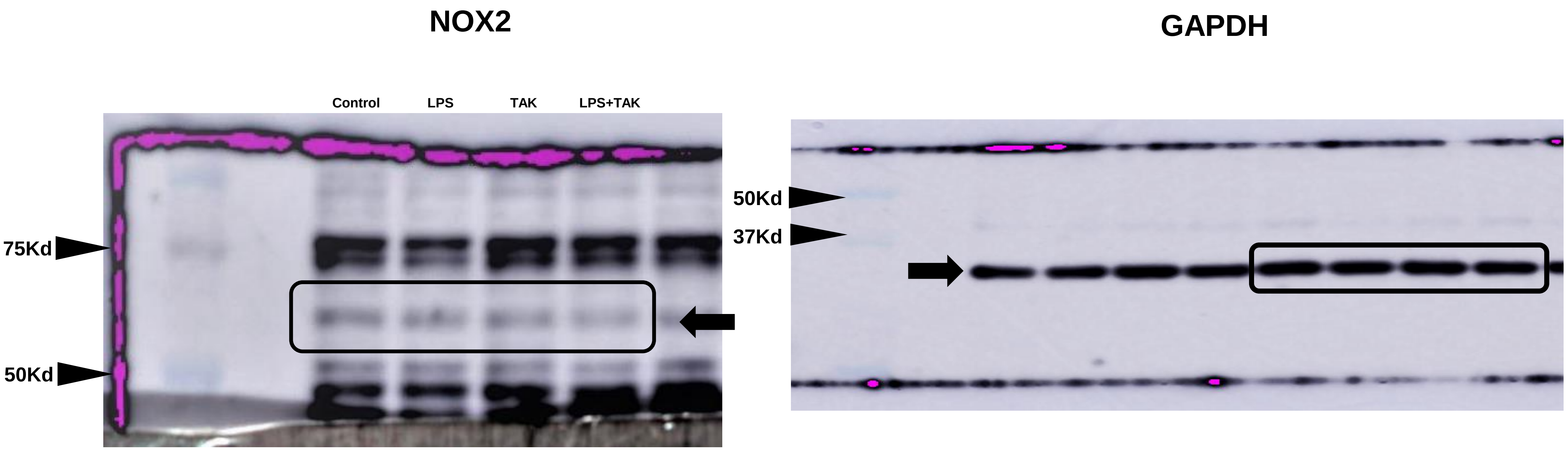

Supplementary Figure 8. Representative full-length immunoblots of Fig. 3E. The amount of NOX2 and GAPDH were shown. The black-line box indicated by arrow in each blot is corresponded to the cropped parts that are showed in the main article.

Supplementary Figure 9

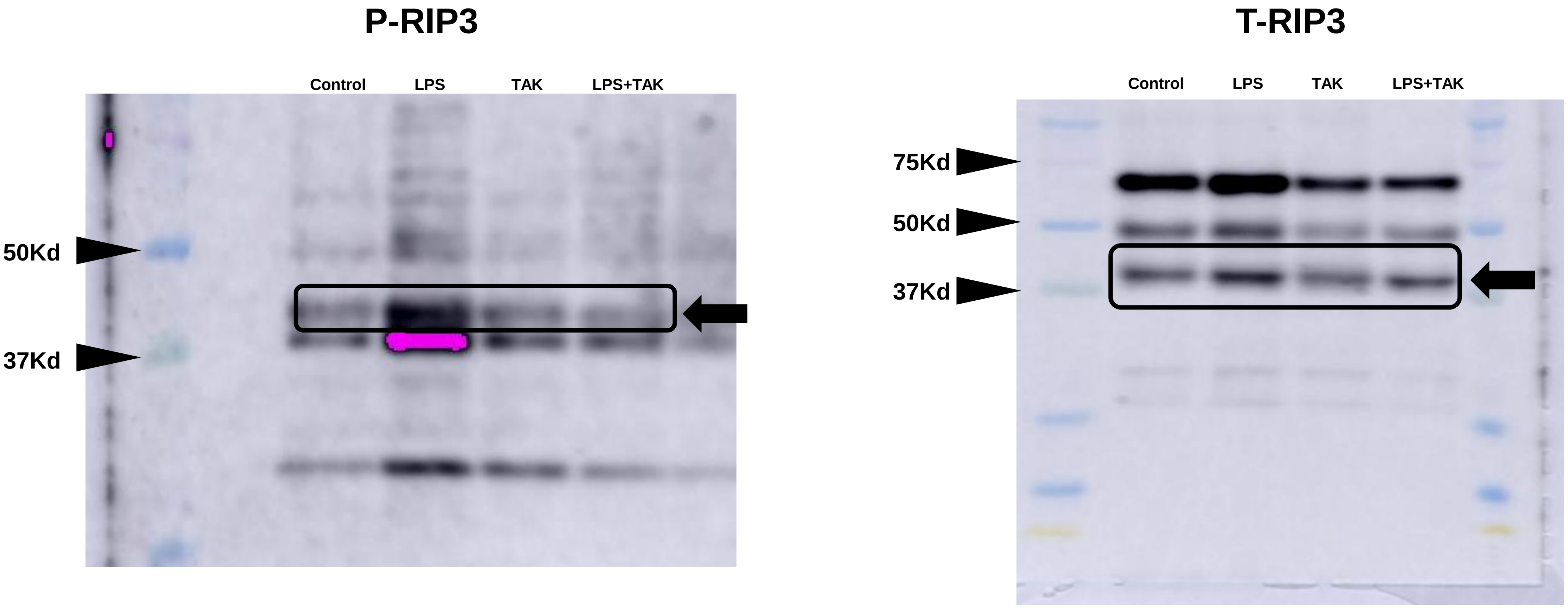

Supplementary Figure 9. Representative full-length immunoblots of Fig. 4A. The amount of P-RIP3 and T-RIP3 were shown. The black-line box indicated by arrow in each blot is corresponded to the cropped parts that are showed in the main article.

Supplementary Figure 10

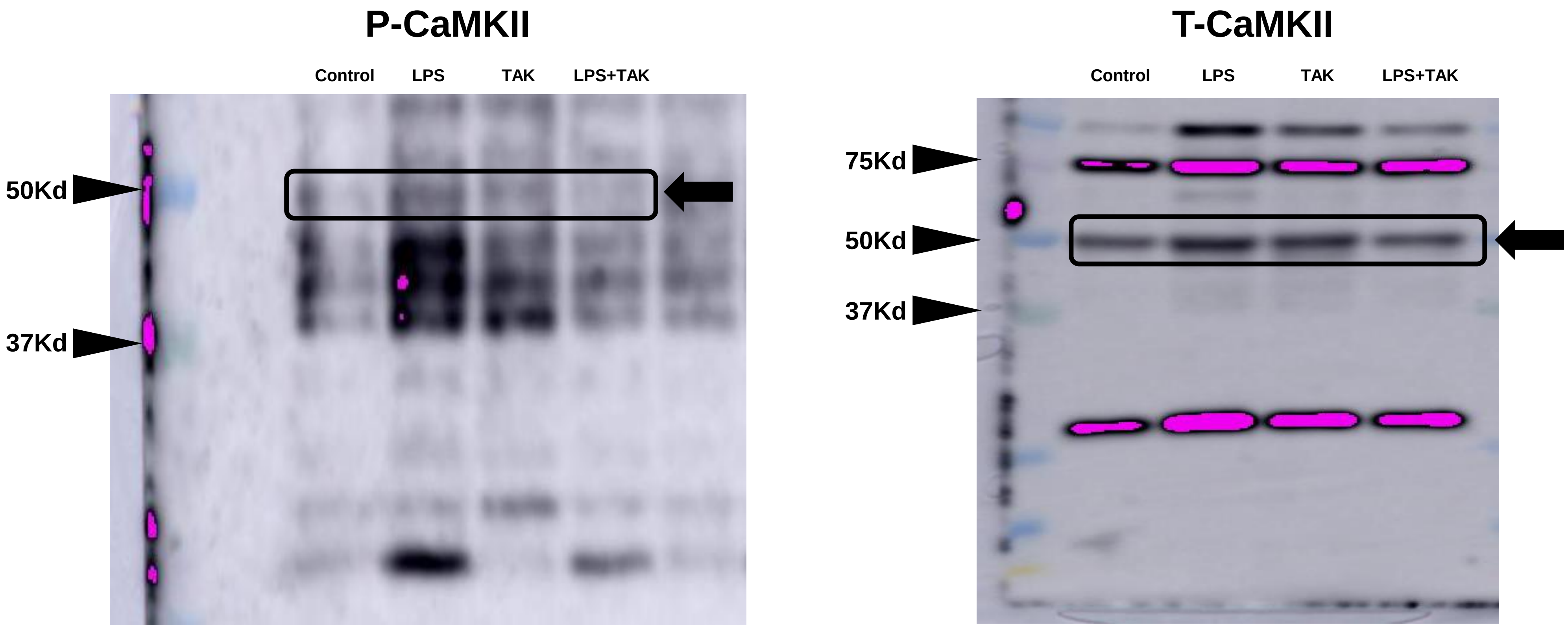

Supplementary Figure 10. Representative full-length immunoblots of Fig. 4B. The amount of P-CaMKII and T-CaMKII were shown. The black-line box indicated by arrow in each blot is corresponded to the cropped parts that are showed in the main article.

Supplementary Figure 11

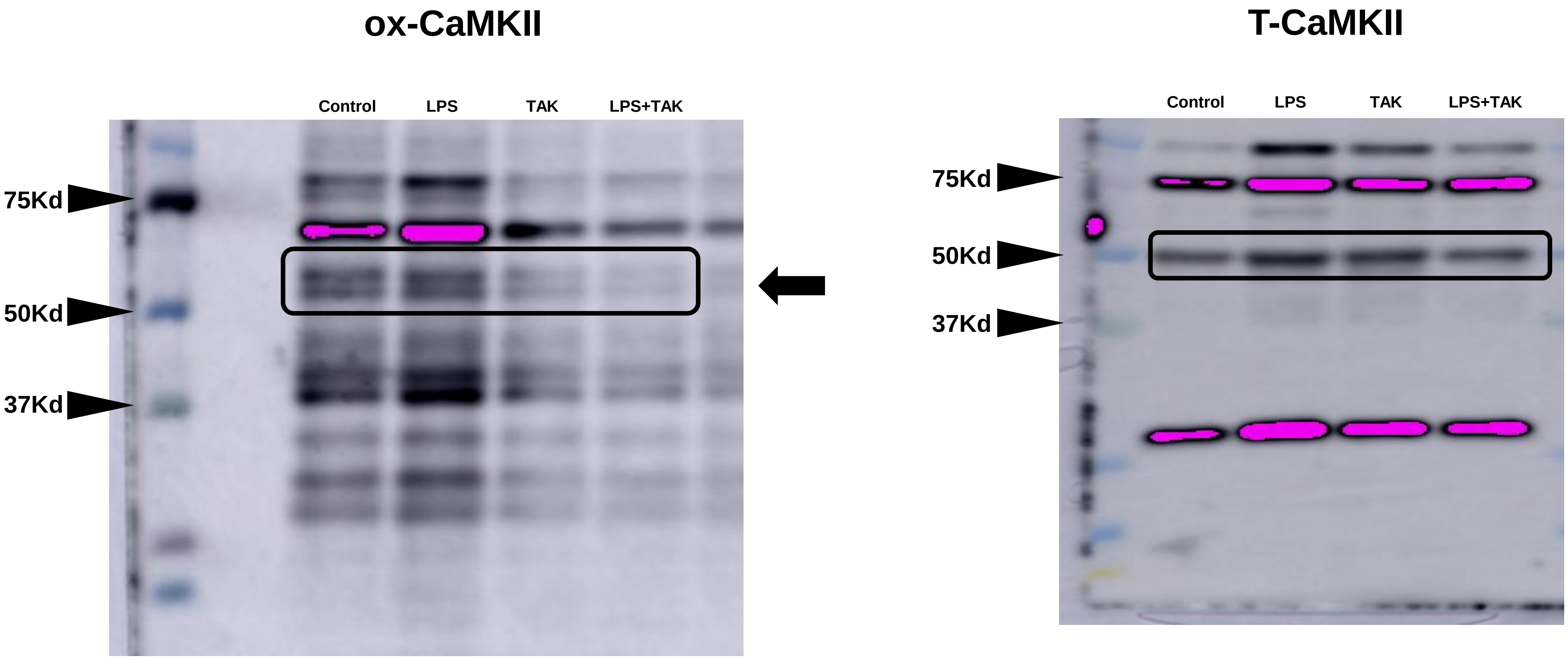

Supplementary Figure 11. Representative full-length immunoblots of Fig. 4C. The amount of ox-CaMKII and T-CaMKII were shown. The black-line box indicated by arrow in each blot is corresponded to the cropped parts that are showed in the main article.

Supplementary Figure 12

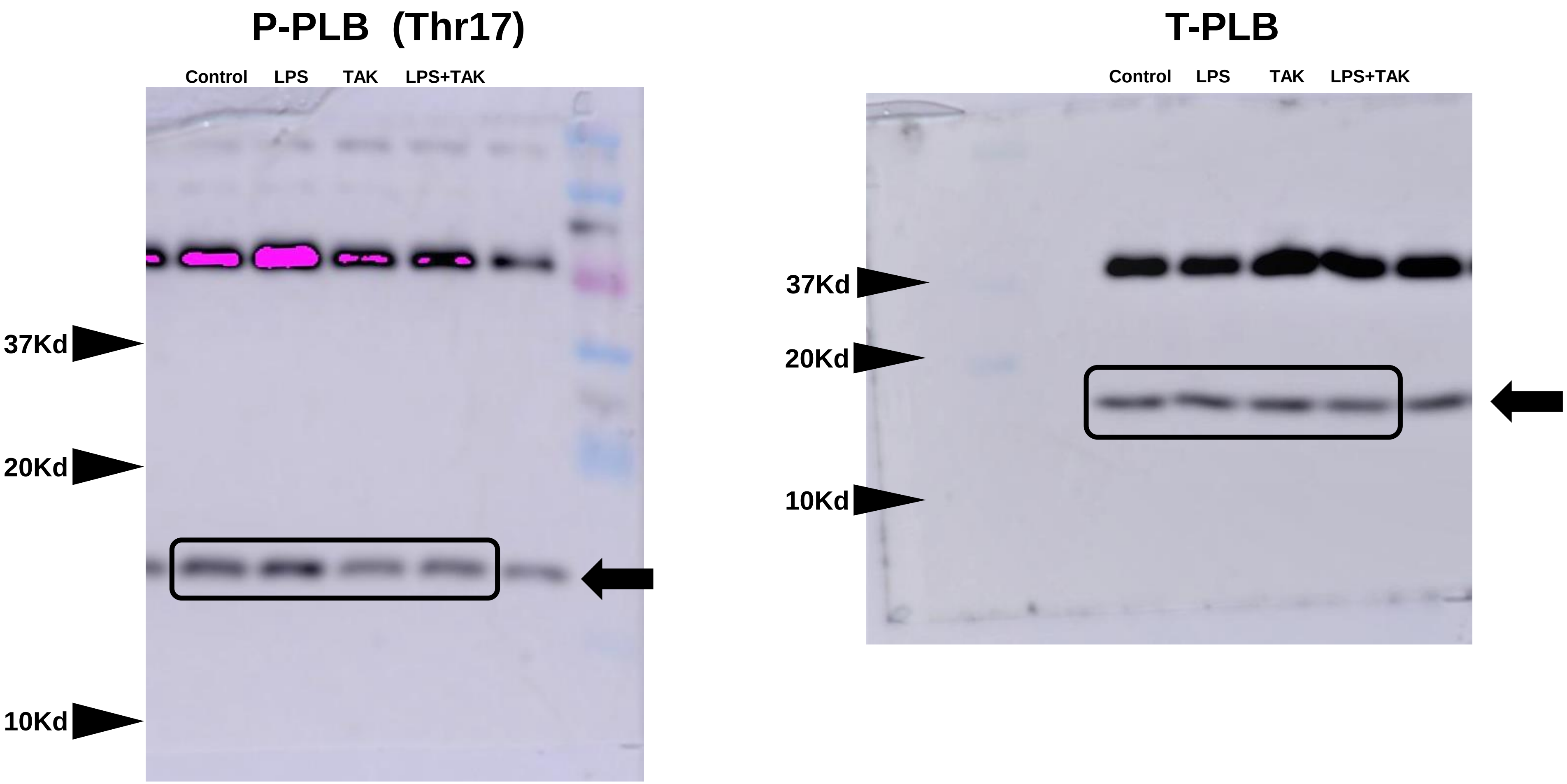

Supplementary Figure 12. Representative full-length immunoblots of Fig. 4D. The amount of P-PLB(Thr17) and T-PLB were shown. The black-line box indicated by arrow in each blot is corresponded to the cropped parts that are showed in the main article.

Supplementary Figure 13

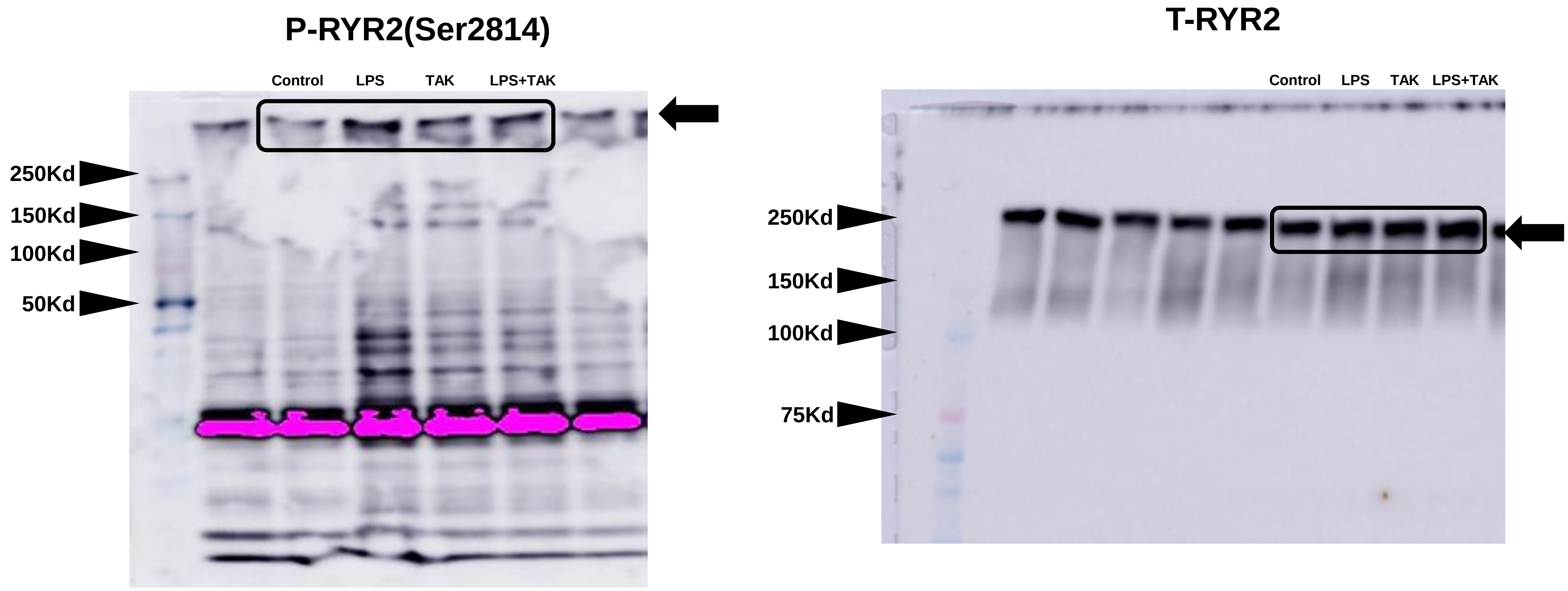

Supplementary Figure 13. Representative full-length immunoblots of Fig. 4E. The amount of P-RYR2(Ser2814) and T-RYR2 were shown. The black-line box indicated by arrow in each blot is corresponded to the cropped parts that are showed in the main article.

Supplementary Figure 14

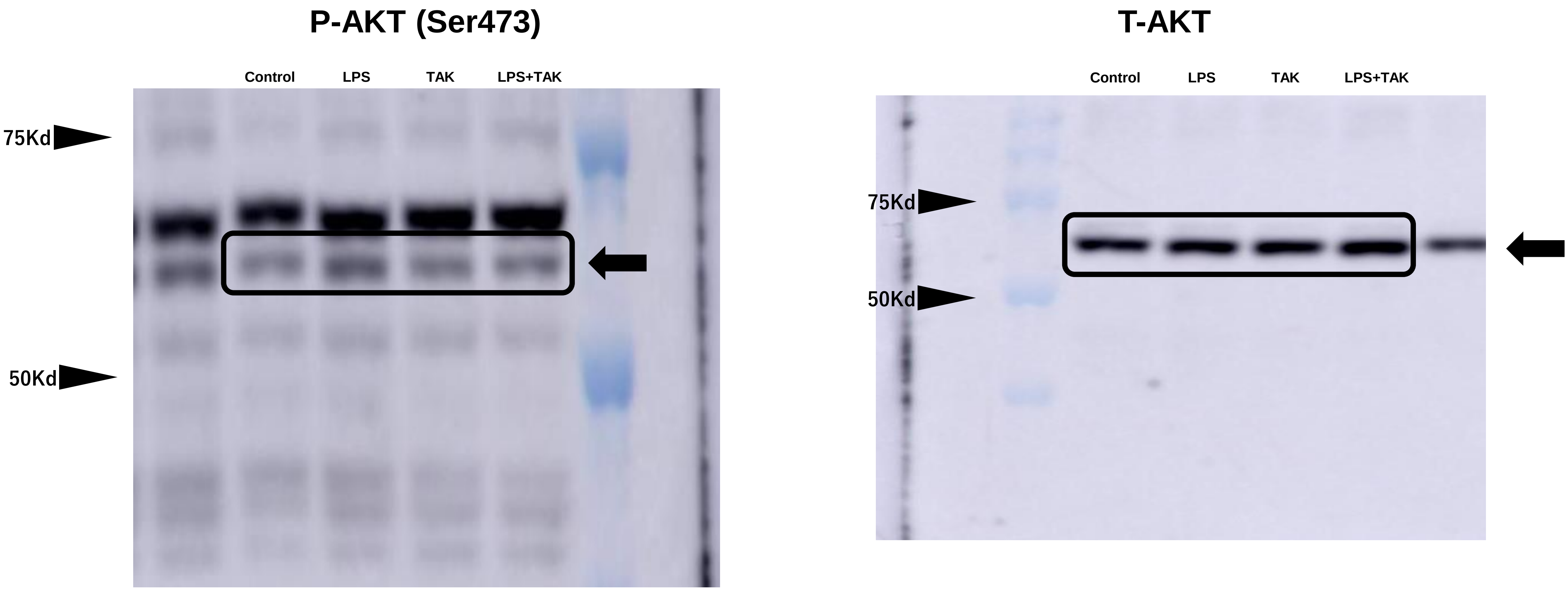

Supplementary Figure 14. Representative full-length immunoblots of Fig. 5A. The amount of P-AKT(Ser473) and T-AKT were shown. The black-line box indicated by arrow in each blot is corresponded to the cropped parts that are showed in the main article.

Supplementary Figure 15

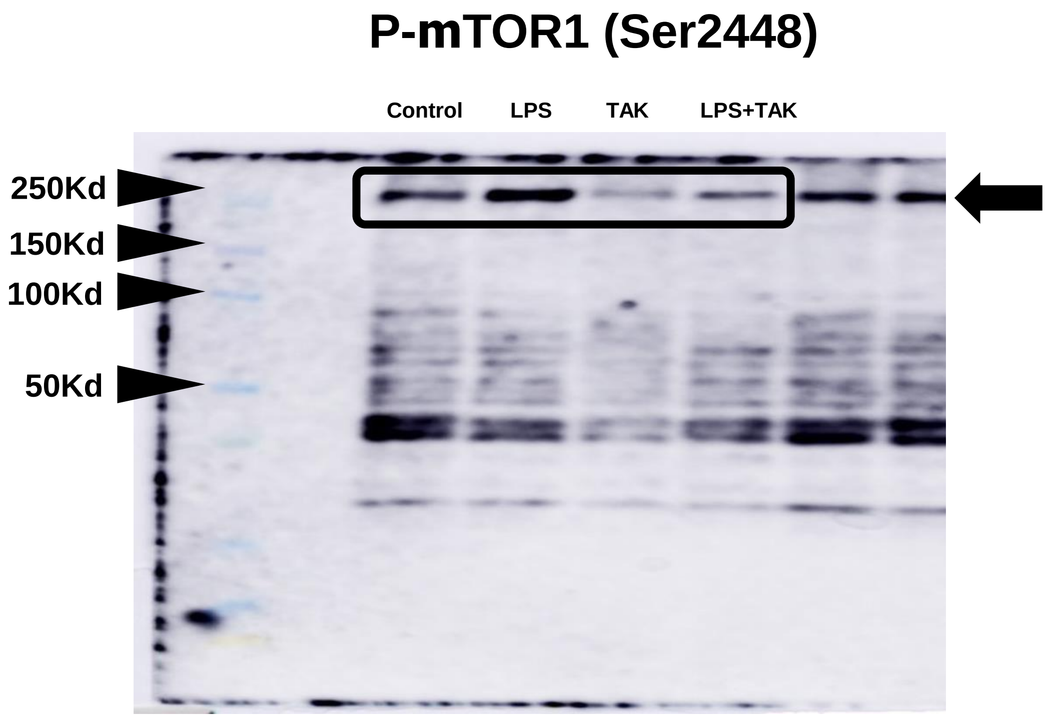

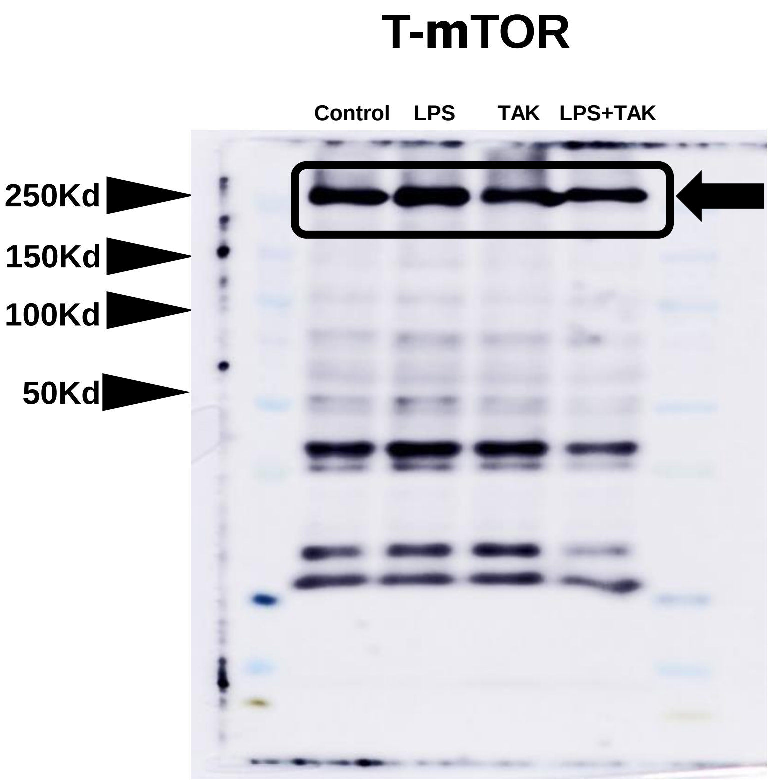

Supplementary Figure 15. Representative full-length immunoblots of Fig. 5B. The amount of P-mTOR1(Ser2448) and T-mTOR were shown. The black-line box indicated by arrow in each blot is corresponded to the cropped parts that are showed in the main article.

Supplementary Figure 16

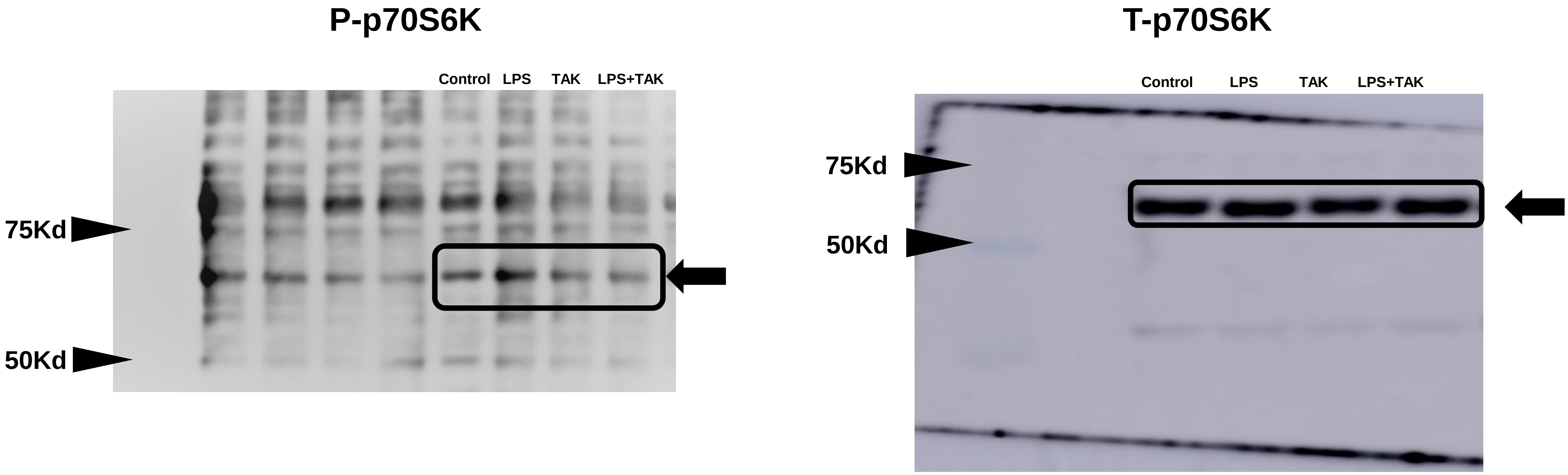

Supplementary Figure 16. Representative full-length immunoblots of Fig. 5C. The amount of P-p70S6K and T- p70S6K were shown. The black-line box indicated by arrow in each blot is corresponded to the cropped parts that are showed in the main article.

Supplementary Figure 17

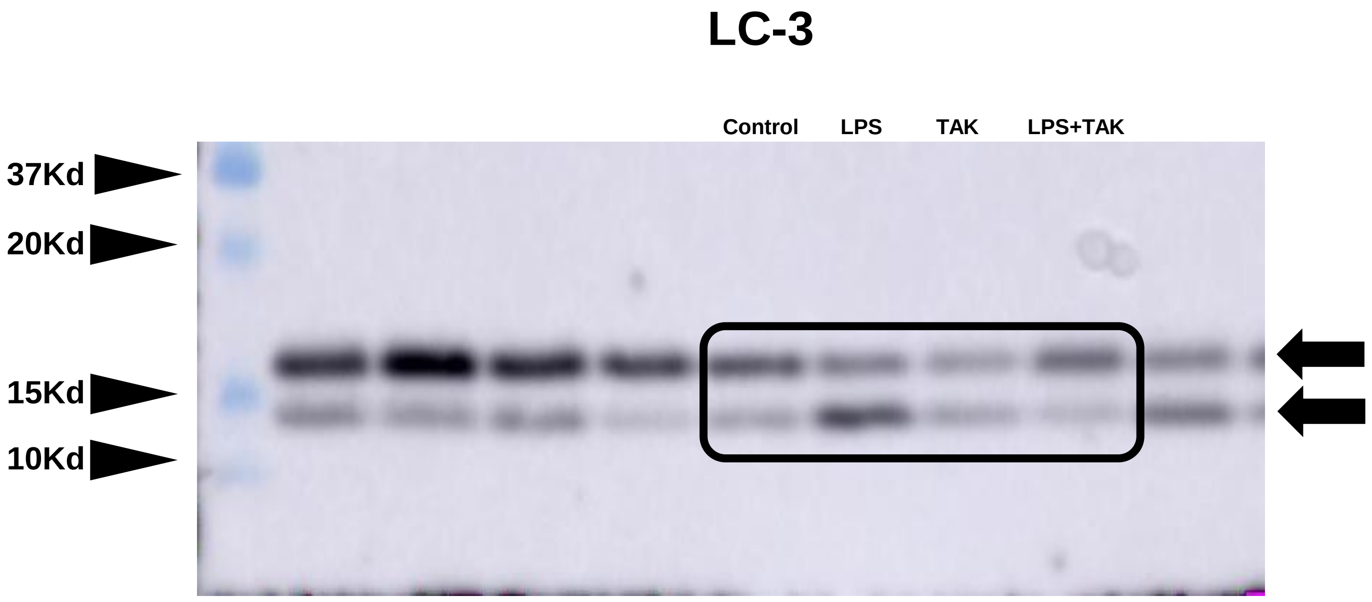

Supplementary Figure 17. Representative full-length immunoblots of Fig. 6A. The amount of LC-3 were shown.
The black-line box indicated by arrow in each blot is corresponded to the cropped parts that are showed in the main article.

Supplementary Figure 18

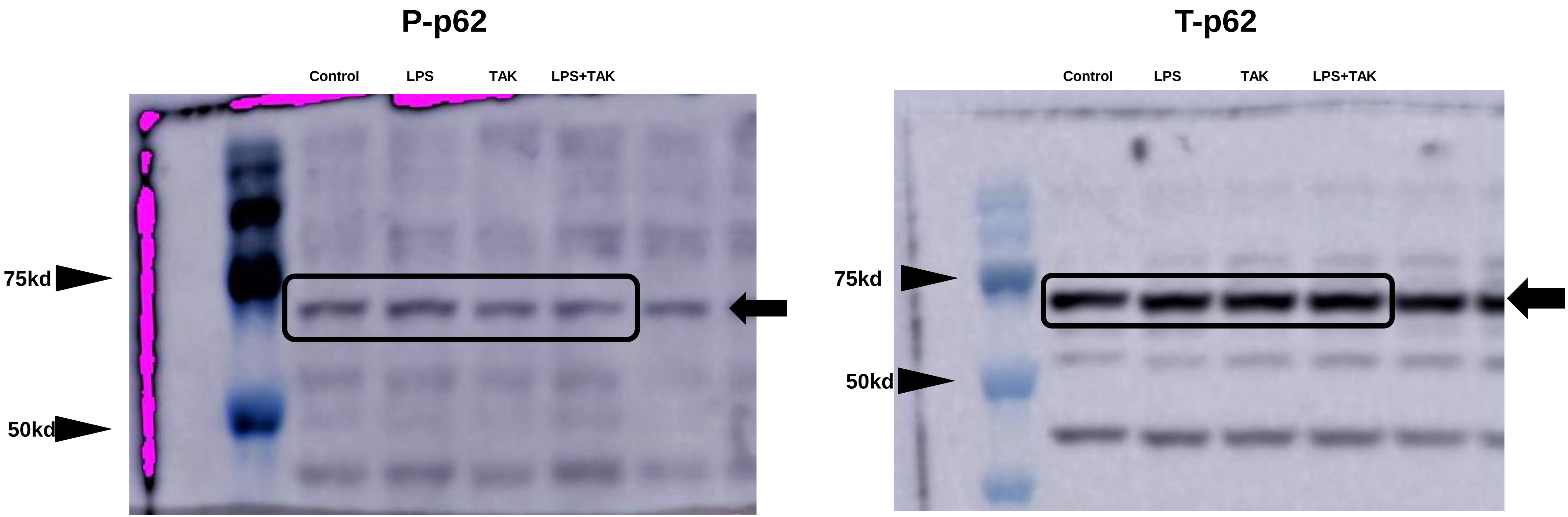

Supplementary Figure 18. Representative full-length immunoblots of Fig. 6A. The amount of P-p62 and T-p62 were shown. The black-line box indicated by arrow in each blot is corresponded to the cropped parts that are showed in the main article.

Supplementary Figure 19

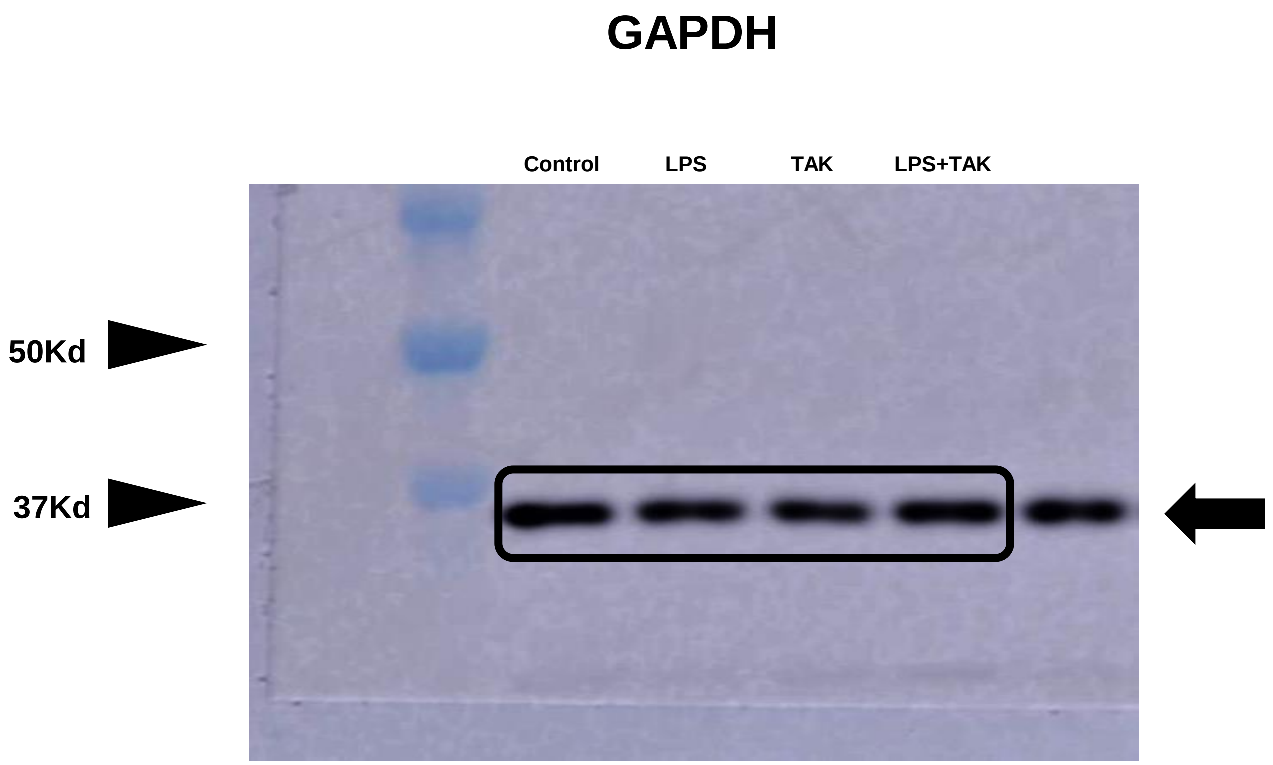

Supplementary Figure 19. Representative full-length immunoblots of Fig. 6A. The amount GAPDH were shown.
The black-line box indicated by arrow in each blot is corresponded to the cropped parts that are showed in the main article.

Supplementary Figure 20

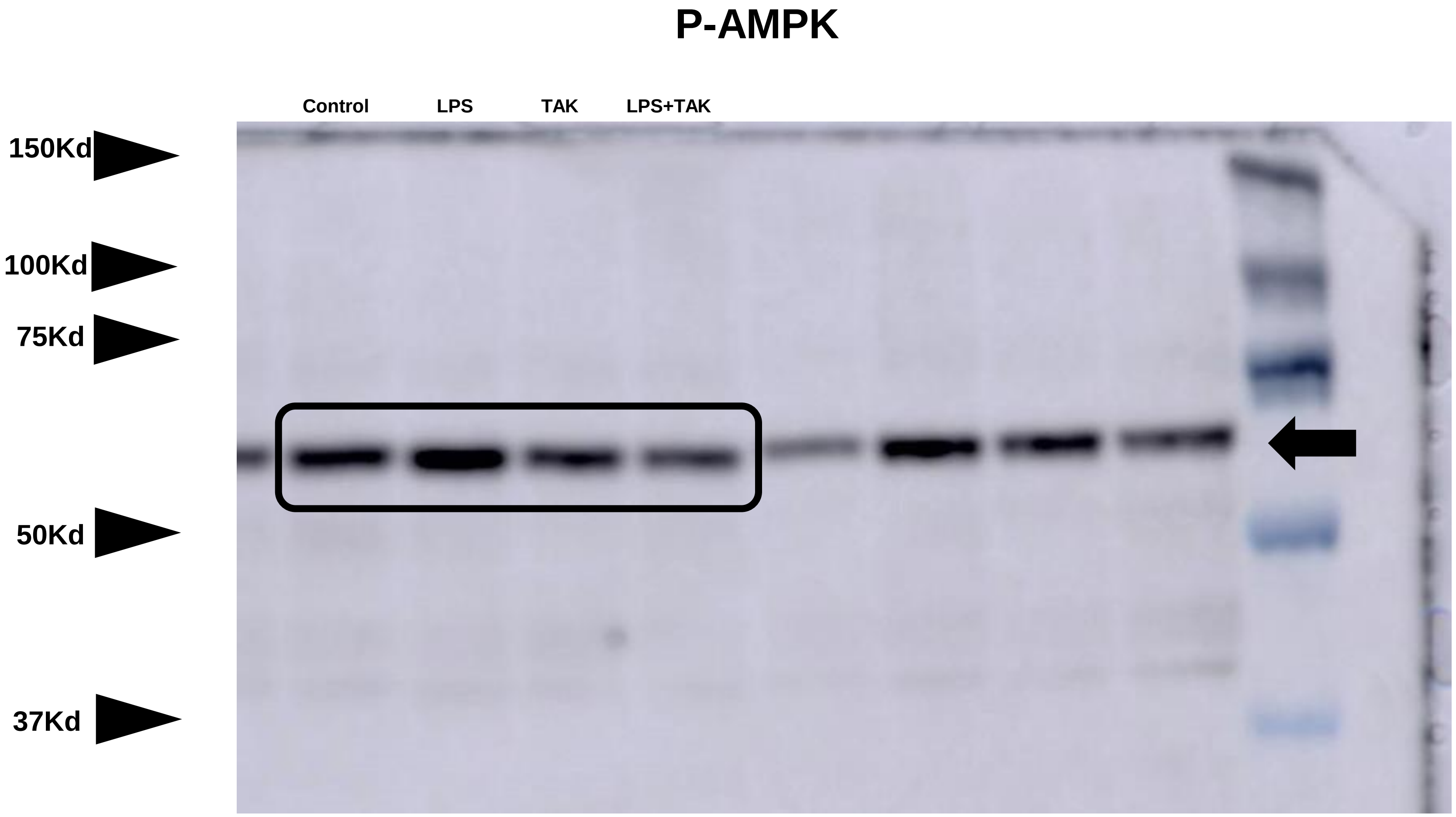

Supplementary Figure 20. Representative full-length immunoblots of Fig. 6A. The amount P-AMPK was shown. The black-line box indicated by arrow in each blot is corresponded to the cropped parts that are showed in the main article.

Supplementary Figure 21

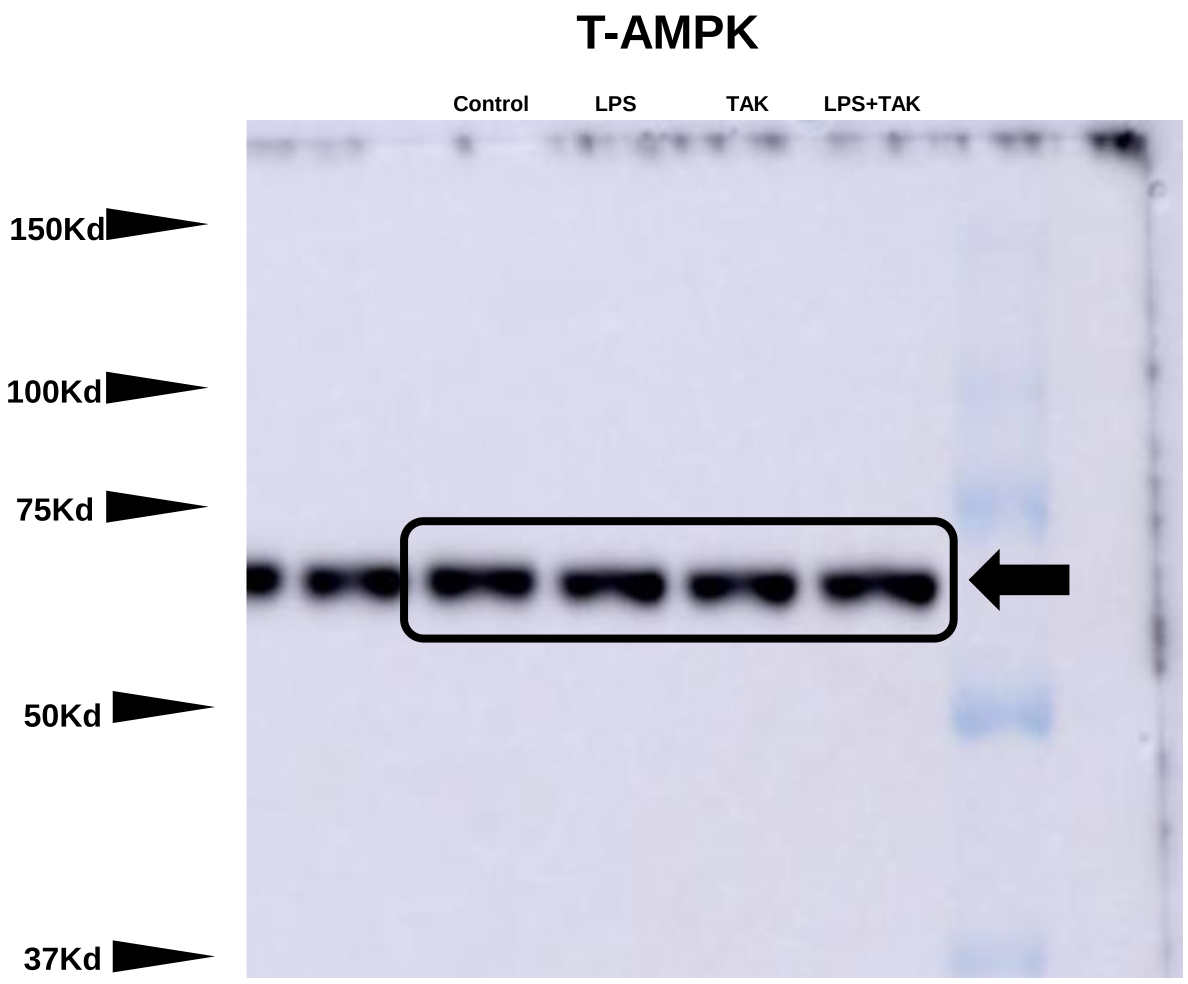

Supplementary Figure 21. Representative full-length immunoblots of Fig.6A. The amount T-AMPK was shown.
The black-line box indicated by arrow in each blot is corresponded to the cropped parts that are showed in the main article.
