# Supplementary material for: Role of TLR4 signaling on Porphyromonas gingivalis LPS-induced cardiac dysfunction in mice
Source: PLoS One. 2022 Jun 1;17(6):e0258823. doi: 10.1371/journal.pone.0258823 (PMC9159598; doi:10.1371/journal.pone.0258823)
Supplement: S2 Data — (PDF) [file pone.0258823.s002.pdf]

Supplementary Figures of S2 Data

Role of TLR4 signaling on *Porphyromonas gingivalis* LPS-induced cardiac dysfunction in mice

Running title: Oxidative stress in the heart of periodontitis

Ichiro Matsuo [1,2], Naoya Kawamura [1,2], Yoshiki Ohnuki [1], Kenji Suita [1], Misao Ishikawa [3], Takehiro Matsubara [4], Yasumasa Mototani [1], Aiko Ito [5], Yoshio Hayakawa [1,6], Megumi Nariyama [7], Akinaka Morii [1,2], Kenichi Kiyomoto [1,2], Michinori Tsunoda [1,2], Kazuhiro Gomi [2], Satoshi Okumura [1]

[1] Department of Physiology, Tsurumi University School of Dental Medicine, Yokohama 230-8501, Japan

[2] Department of Periodontology, Tsurumi University School of Dental Medicine, Yokohama 230-8501, Japan

[3] Department of Oral Anatomy, Tsurumi University School of Dental Medicine, Yokohama 230-8501, Japan

[4] Division of BioBank, Center for Comprehensive Genomic Medicine, Okayama University Hospital, Okayama, Japan

[5] Department of Orthodontics, Tsurumi University School of Dental Medicine, Yokohama 230-8501, Japan

[6] Department of Dental Anesthesiology, Tsurumi University School of Dental Medicine, Yokohama 230-8501, Japan

[7] Department of Pediatric Dentistry, Tsurumi University School of Dental Medicine, Yokohama 236-8501, Japan

*****Corresponding author: Satoshi Okumura:

Department of Physiology, Tsurumi University School of Dental Medicine,

2-1-3 Tsurumi, Tsurumi-ku, Yokohama 230-8501; (Tel. +81-(0)45-580-8476;

Fax. +81-(0)45-585-2889; e-mail: okumura-s@tsurumi-u.ac.jp)

Supplementary Figure 1

A

**TNF-α**		*P* Value (Tukey)
Control N=7	LPS	0.7178
	TAK	1.0000
	LPS+TAK	0.1274
LPS N=7	Control	0.7178
	TAK	0.7350
	LPS+TAK	0.6101
TAK N=7	Control	1.0000
	LPS	0.7350
	LPS+TAK	0.1347
LPS+TAK N=7	Control	0.1274
	LPS	0.1274
	TAK	0.1347

One way ANOVA followed by Tukey-Kramer *post hoc* test

B

**IL-1β**		*P* Value (Tukey)
Control N=7	LPS	0.9990
	TAK	0.8512
	LPS+TAK	1.0000
LPS N=7	Control	0.9990
	TAK	0.7808
	LPS+TAK	0.9972
TAK N=7	Control	0.8512
	LPS	0.7808
	LPS+TAK	0.8768
LPS+TAK N=7	Control	1.0000
	LPS	0.9972
	TAK	0.8768

One way ANOVA followed by Tukey-Kramer *post hoc* test

Supplementary Figure 1.
A: Statistical analysis of Table1 (TNF-α)
B: Statistical analysis of Table1 (IL-1β)

Supplementary Figure 2

A

IL-6		*P* Value (Tukey)
Control N=7	LPS	0.6197
	TAK	0.9998
	LPS+TAK	0.4010
LPS N=7	Control	0.6197
	TAK	0.5669
	LPS+TAK	0.9820
TAK N=7	Control	0.9998
	LPS	0.5669
	LPS+TAK	0.3555
LPS+TAK N=7	Control	0.4010
	LPS	0.9820
	TAK	0.3555

One way ANOVA followed by Tukey-Kramer *post hoc* test

B

IL-10		*P* Value (Tukey)
Control N=7	LPS	0.4616
	TAK	0.5542
	LPS+TAK	0.4967
LPS N=7	Control	0.4616
	TAK	0.0444
	LPS+TAK	1.0000
TAK N=7	Control	0.5542
	LPS	0.0444
	LPS+TAK	0.0505
LPS+TAK N=7	Control	0.4967
	LPS	1.0000
	TAK	0.0505

One way ANOVA followed by Tukey-Kramer *post hoc* test

Supplementary Figure 2.
A: Statistical analysis of Table1 (IL-6)
B: Statistical analysis of Table1 (IL-10)

Supplementary Figure 3

A

**IL-17A**		*P* Value (Tukey)
Control N=7	LPS	0.9992
	TAK	0.7200
	LPS+TAK	0.4368
LPS N=7	Control	0.9992
	TAK	0.6433
	LPS+TAK	0.3673
TAK N=7	Control	0.7200
	LPS	0.6433
	LPS+TAK	0.9639
LPS+TAK N=7	Control	0.4368
	LPS	0.3673
	TAK	0.9639

One way ANOVA followed by Tukey-Kramer *post hoc* test

B

**IFN-γ**		*P* Value (Tukey)
Control N=7	LPS	0.9987
	TAK	0.4265
	LPS+TAK	0.9758
LPS N=7	Control	0.9987
	TAK	0.3455
	LPS+TAK	0.9937
TAK N=7	Control	0.4265
	LPS	0.3455
	LPS+TAK	0.2309
LPS+TAK N=7	Control	0.9758
	LPS	0.9937
	TAK	0.2309

One way ANOVA followed by Tukey-Kramer *post hoc* test

Supplementary Figure 3.
A: Statistical analysis of Table1 (IL-17A)
B: Statistical analysis of Table 1 (IFN- γ)

Supplementary Figure 4

A

**MCP-1**		*P* Value (Tukey)
Control N=7	LPS	0.9996
	TAK	0.9996
	LPS+TAK	0.9996
LPS N=7	Control	0.9996
	TAK	1.0000
	LPS+TAK	0.9965
TAK N=7	Control	0.9996
	LPS	1.0000
	LPS+TAK	0.9967
LPS+TAK N=7	Control	0.9996
	LPS	0.9965
	TAK	0.9967

One way ANOVA followed by Tukey-Kramer *post hoc* test

B

**VEGF-A**		*P* Value (Tukey)
Control N=7	LPS	0.9997
	TAK	0.7789
	LPS+TAK	0.9985
LPS N=7	Control	0.9997
	TAK	0.7254
	LPS+TAK	0.9999
TAK N=7	Control	0.6867
	LPS	0.7254
	LPS+TAK	0.6967
LPS+TAK N=7	Control	0.9985
	LPS	0.9999
	TAK	0.6967

One way ANOVA followed by Tukey-Kramer *post hoc* test

Supplementary Figure 4.
A: Statistical analysis of Table 1 (MCP-1)
B: Statistical analysis of Table 1 (VEGF-A)

Supplementary Figure 5

A

**PDGF-BB**		*P* Value (Tukey)
Control N=7	LPS	0.9967
	TAK	0.9912
	LPS+TAK	0.9779
LPS N=7	Control	0.9967
	TAK	0.9587
	LPS+TAK	0.9974
TAK N=7	Control	0.9912
	LPS	0.9587
	LPS+TAK	0.9000
LPS+TAK N=7	Control	0.9779
	LPS	0.9974
	TAK	0.9000

One way ANOVA followed by Tukey-Kramer *post hoc* test

Supplementary Figure 5.
A: Statistical analysis of Table 1 (PDGF-BB)

Supplementary Figure 6

A

**EF 1week**		*P* Value (Tukey)
Control N=7	LPS	0.0001
	TAK	0.0045
	LPS+TAK	0.0001
LPS N=7	Control	0.0001
	TAK	0.0018
	LPS+TAK	0.1516
TAK N=7	Control	0.0045
	LPS	0.0018
	LPS+TAK	0.2246
LPS+TAK N=7	Control	0.0001
	LPS	0.1516
	TAK	0.2246

One way ANOVA followed by Tukey-Kramer *post hoc* test

B

**LVEDV 1week**		*P* Value (Tukey)
Control N=7	LPS	0.8166
	TAK	0.9631
	LPS+TAK	0.3534
LPS N=7	Control	0.8166
	TAK	0.9781
	LPS+TAK	0.8503
TAK N=7	Control	0.9631
	LPS	0.9781
	LPS+TAK	0.6293
LPS+TAK N=7	Control	0.3534
	LPS	0.8503
	TAK	0.6293

One way ANOVA followed by Tukey-Kramer *post hoc* test

Supplementary Figure 6.
A: Statistical analysis of Table 2A (EF 1w)
B: Statistical analysis of Table 2A (LVEDV 1w)

Supplementary Figure 7

A

**LVESV 1week**		*P* Value (Tukey)
Control N=7	LPS	0.0113
	TAK	0.3808
	LPS+TAK	0.0088
LPS N=7	Control	0.0113
	TAK	0.3032
	LPS+TAK	0.9996
TAK N=7	Control	0.3808
	LPS	0.3032
	LPS+TAK	0.2587
LPS+TAK N=7	Control	0.0088
	LPS	0.9996
	TAK	0.2587

One way ANOVA followed by Tukey-Kramer *post hoc* test

B

**FS 1week**		*P* Value (Tukey)
Control N=7	LPS	0.0001
	TAK	0.0026
	LPS+TAK	0.0001
LPS N=7	Control	0.0001
	TAK	0.0023
	LPS+TAK	0.1616
TAK N=7	Control	0.0026
	LPS	0.0023
	LPS+TAK	0.2536
LPS+TAK N=7	Control	0.0001
	LPS	0.1616
	TAK	0.2536

One way ANOVA followed by Tukey-Kramer *post hoc* test

Supplementary Figure 7.
A: Statistical analysis of Table 2A (LVESV 1w)
B: Statistical analysis of Table 2A (FS 1w)

Supplementary Figure 8

A

**LVDd 1week**		*P* Value (Tukey)
Control N=7	LPS	0.8044
	TAK	0.9490
	LPS+TAK	0.3299
LPS N=7	Control	0.8044
	TAK	0.9832
	LPS+TAK	0.8393
TAK N=7	Control	0.9490
	LPS	0.9832
	LPS+TAK	0.6362
LPS+TAK N=7	Control	0.3299
	LPS	0.8393
	TAK	0.6362

One way ANOVA followed by Tukey-Kramer *post hoc* test

B

**LVDs 1week**		*P* Value (Tukey)
Control N=7	LPS	0.0084
	TAK	0.3134
	LPS+TAK	0.0073
LPS N=7	Control	0.0084
	TAK	0.3092
	LPS+TAK	1.0000
TAK N=7	Control	0.3134
	LPS	0.3092
	LPS+TAK	0.2811
LPS+TAK N=7	Control	0.0073
	LPS	1.0000
	TAK	0.2811

One way ANOVA followed by Tukey-Kramer *post hoc* test

Supplementary Figure 8.
A: Statistical analysis of Table 2A (LVDd 1w)
B: Statistical analysis of Table 2A (LVDs 1w)

Supplementary Figure 9

A

**HR 1week**		*P* Value (Tukey)
Control N=7	LPS	1.0000
	TAK	0.9703
	LPS+TAK	0.7073
LPS N=7	Control	1.0000
	TAK	0.9767
	LPS+TAK	0.7284
TAK N=7	Control	0.9703
	LPS	0.9767
	LPS+TAK	0.9205
LPS+TAK N=7	Control	0.7073
	LPS	0.7284
	TAK	0.9205

One way ANOVA followed by Tukey-Kramer *post hoc* test

B

**SV 1week**		*P* Value (Tukey)
Control N=7	LPS	0.8264
	TAK	0.9892
	LPS+TAK	0.9852
LPS N=7	Control	0.8264
	TAK	0.9469
	LPS+TAK	0.6297
TAK N=7	Control	0.9892
	LPS	0.9469
	LPS+TAK	0.9110
LPS+TAK N=7	Control	0.9852
	LPS	0.6297
	TAK	0.9110

One way ANOVA followed by Tukey-Kramer *post hoc* test

Supplementary Figure 9.
A: Statistical analysis of Table 2A (HR 1w)
B: Statistical analysis of Table 2A (SV 1w)

Supplementary Figure 10

A

**CO 1week**		*P* Value (Tukey)
Control<br>N=7	LPS	0.8038
	TAK	0.6497
	LPS+TAK	0.8647
LPS<br>N=7	Control	0.8038
	TAK	0.9997
	LPS+TAK	0.9993
TAK<br>N=7	Control	0.6497
	LPS	0.9997
	LPS+TAK	1.0000
LPS+TAK<br>N=7	Control	0.8647
	LPS	0.9993
	TAK	1.0000

One way ANOVA followed by Tukey-Kramer *post hoc* test

B

**IVSTd 1week**		*P* Value (Tukey)
Control<br>N=7	LPS	0.9996
	TAK	0.7672
	LPS+TAK	0.9423
LPS<br>N=7	Control	0.9996
	TAK	0.7068
	LPS+TAK	0.9678
TAK<br>N=7	Control	0.7672
	LPS	0.7068
	LPS+TAK	0.4346
LPS+TAK<br>N=7	Control	0.9423
	LPS	0.9678
	TAK	0.4346

One way ANOVA followed by Tukey-Kramer *post hoc* test

Supplementary Figure 10.
A: Statistical analysis of Table 2A (CO 1w)
B: Statistical analysis of Table 2A (IVSTd 1w)

Supplementary Figure 11

A

**IVSTs 1week**		*P* Value (Tukey)
Control N=7	LPS	0.3061
	TAK	0.3026
	LPS+TAK	0.5011
LPS N=7	Control	0.3060
	TAK	1.0000
	LPS+TAK	0.9830
TAK N=7	Control	0.3026
	LPS	1.0000
	LPS+TAK	0.9820
LPS+TAK N=7	Control	0.5011
	LPS	0.9830
	TAK	0.9820

One way ANOVA followed by Tukey-Kramer *post hoc* test

B

**PWTd 1week**		*P* Value (Tukey)
Control N=7	LPS	1.0000
	TAK	1.0000
	LPS+TAK	0.9984
LPS N=7	Control	1.0000
	TAK	1.0000
	LPS+TAK	0.9971
TAK N=7	Control	1.0000
	LPS	1.0000
	LPS+TAK	0.9984
LPS+TAK N=7	Control	0.9984
	LPS	0.9971
	TAK	0.9984

One way ANOVA followed by Tukey-Kramer *post hoc* test

Supplementary Figure 11.
A: Statistical analysis of Table 2A (IVSTs 1w)
B: Statistical analysis of Table 2A (PWTd 1w)

Supplementary Figure 12

A

PWTs 1week		*P* Value (Tukey)
Control<br>N=7	LPS	0.2934
	TAK	0.9977
	LPS+TAK	0.9696
LPS<br>N=7	Control	0.2934
	TAK	0.2182
	LPS+TAK	0.5337
TAK<br>N=7	Control	0.9977
	LPS	0.2182
	LPS+TAK	0.9211
LPS+TAK<br>N=7	Control	0.9696
	LPS	0.5337
	TAK	0.9211

One way ANOVA followed by Tukey-Kramer *post hoc* test

Supplementary Figure 12.
A: Statistical analysis of Table 2A (PWTs 1w)

Supplementary Figure 13

A

**EF 4week**		*P* Value (Tukey)
Control N=7	LPS	0.0001
	TAK	0.0004
	LPS+TAK	0.0037
LPS N=7	Control	0.0001
	TAK	0.1603
	LPS+TAK	0.0249
TAK N=7	Control	0.0004
	LPS	0.1603
	LPS+TAK	0.8025
LPS+TAK N=7	Control	0.0037
	LPS	0.0249
	TAK	0.8025

One way ANOVA followed by Tukey-Kramer *post hoc* test

B

**LVEDV 4week**		*P* Value (Tukey)
Control N=7	LPS	0.9751
	TAK	0.7496
	LPS+TAK	0.7811
LPS N=7	Control	0.9751
	TAK	0.9354
	LPS+TAK	0.9513
TAK N=7	Control	0.7496
	LPS	0.9354
	LPS+TAK	1.0000
LPS+TAK N=7	Control	0.7811
	LPS	0.9513
	TAK	1.0000

One way ANOVA followed by Tukey-Kramer *post hoc* test

Supplementary Figure 13.
A: Statistical analysis of Table 2B (EF 4w)
B: Statistical analysis of Table 2B (LVEDV 4w)

Supplementary Figure 14

A

**LVESV 4week**		*P* Value (Tukey)
Control N=7	LPS	0.1077
	TAK	0.1199
	LPS+TAK	0.2627
LPS N=7	Control	0.1077
	TAK	1.0000
	LPS+TAK	0.9578
TAK N=7	Control	0.1199
	LPS	1.0000
	LPS+TAK	0.9697
LPS+TAK N=7	Control	0.2627
	LPS	0.9578
	TAK	0.9697

One way ANOVA followed by Tukey-Kramer *post hoc* test

B

**FS 4week**		*P* Value (Tukey)
Control N=7	LPS	0.0001
	TAK	0.0003
	LPS+TAK	0.0023
LPS N=7	Control	0.0001
	TAK	0.1493
	LPS+TAK	0.0281
TAK N=7	Control	0.0003
	LPS	0.1493
	LPS+TAK	0.8477
LPS+TAK N=7	Control	0.0003
	LPS	0.0281
	TAK	0.8477

One way ANOVA followed by Tukey-Kramer *post hoc* test

Supplementary Figure 14.
A: Statistical analysis of Table 2B (LVESV 4w)
B: Statistical analysis of Table 2B (FS 4w)

Supplementary Figure 15

A

**LVDd 4week**		*P* Value (Tukey)
Control N=7	LPS	0.9893
	TAK	0.8112
	LPS+TAK	0.7956
LPS N=7	Control	0.9893
	TAK	0.9381
	LPS+TAK	0.9289
TAK N=7	Control	0.8112
	LPS	0.9381
	LPS+TAK	1.0000
LPS+TAK N=7	Control	0.7956
	LPS	0.9289
	TAK	1.0000

One way ANOVA followed by Tukey-Kramer *post hoc* test

B

**LVDs 4week**		*P* Value (Tukey)
Control N=7	LPS	0.0746
	TAK	0.0978
	LPS+TAK	0.3066
LPS N=7	Control	0.0746
	TAK	0.9991
	LPS+TAK	0.8600
TAK N=7	Control	0.0978
	LPS	0.9991
	LPS+TAK	0.9142
LPS+TAK N=7	Control	0.3066
	LPS	0.8600
	TAK	0.9142

One way ANOVA followed by Tukey-Kramer *post hoc* test

Supplementary Figure 15.
A: Statistical analysis of Table 2B (LVDd 4w)
B: Statistical analysis of Table 2B (LVDs 4w)

Supplementary Figure 16

A

**HR 4week**		*P* Value (Tukey)
Control	LPS	0.9996
N=7	TAK	0.7004
	LPS+TAK	0.9709
LPS	Control	0.9996
N=7	TAK	0.6413
	LPS+TAK	0.9487
TAK	Control	0.7004
N=7	LPS	0.6413
	LPS+TAK	0.9155
LPS+TAK	Control	0.9709
N=7	LPS	0.9487
	TAK	0.9155

One way ANOVA followed by Tukey-Kramer *post hoc* test

B

**SV 4week**		*P* Value (Tukey)
Control	LPS	0.6092
N=7	TAK	0.9986
	LPS+TAK	0.9995
LPS	Control	0.6092
N=7	TAK	0.7039
	LPS+TAK	0.5412
TAK	Control	0.9986
N=7	LPS	0.7039
	LPS+TAK	0.9930
LPS+TAK	Control	0.9995
N=7	LPS	0.5412
	TAK	0.9930

One way ANOVA followed by Tukey-Kramer *post hoc* test

Supplementary Figure 16.
A: Statistical analysis of Table 2B (HR 4w)
B: Statistical analysis of Table 2B (SV 4w)

Supplementary Figure 17

A

**CO 4week**		*P* Value (Tukey)
Control N=7	LPS	0.7633
	TAK	0.8464
	LPS+TAK	0.9919
LPS N=7	Control	0.7633
	TAK	0.9984
	LPS+TAK	0.8979
TAK N=7	Control	0.8464
	LPS	0.9984
	LPS+TAK	0.9501
LPS+TAK N=7	Control	0.9919
	LPS	0.8979
	TAK	0.9501

One way ANOVA followed by Tukey-Kramer *post hoc* test

B

**IVSTd 4week**		*P* Value (Tukey)
Control N=7	LPS	0.9930
	TAK	0.9949
	LPS+TAK	0.9490
LPS N=7	Control	0.9930
	TAK	0.9558
	LPS+TAK	0.8509
TAK N=7	Control	0.9949
	LPS	0.9558
	LPS+TAK	0.9908
LPS+TAK N=7	Control	0.9490
	LPS	0.8509
	TAK	0.9908

One way ANOVA followed by Tukey-Kramer *post hoc* test

Supplementary Figure 17.
A: Statistical analysis of Table 2B (CO 4w)
B: Statistical analysis of Table 2B (IVSTd 4w)

Supplementary Figure 18

A

**IVSTs 4week**		*P* Value (Tukey)
Control<br>N=7	LPS	0.1572
	TAK	0.4340
	LPS+TAK	0.6164
LPS<br>N=7	Control	0.1572
	TAK	0.9154
	LPS+TAK	0.7761
TAK<br>N=7	Control	0.4340
	LPS	0.9154
	LPS+TAK	0.9897
LPS+TAK<br>N=7	Control	0.6164
	LPS	0.7761
	TAK	0.9897

One way ANOVA followed by Tukey-Kramer *post hoc* test

B

**PWTd 4week**		*P* Value (Tukey)
Control<br>N=7	LPS	0.9944
	TAK	0.9369
	LPS+TAK	0.3662
LPS<br>N=7	Control	0.9944
	TAK	0.8398
	LPS+TAK	0.2512
TAK<br>N=7	Control	0.9369
	LPS	0.8398
	LPS+TAK	0.7056
LPS+TAK<br>N=7	Control	0.3662
	LPS	0.2512
	TAK	0.7056

One way ANOVA followed by Tukey-Kramer *post hoc* test

Supplementary Figure 18.
A: Statistical analysis of Table 2B (IVSTs 4w)
B: Statistical analysis of Table 2B (PWTd 4w)

Supplementary Figure 19

A

PWTs 4week		*P* Value (Tukey)
Control N=7	LPS	0.4906
	TAK	0.9762
	LPS+TAK	0.9983
LPS N=7	Control	0.4906
	TAK	0.7366
	LPS+TAK	0.3959
TAK N=7	Control	0.9762
	LPS	0.7366
	LPS+TAK	0.9378
LPS+TAK N=7	Control	0.9983
	LPS	0.3959
	TAK	0.9378

One way ANOVA followed by Tukey-Kramer *post hoc* test

Supplementary Figure 19.
A: Statistical analysis of Table 2B (PWTs 4w)
