# Supplementary material for: Role of TLR4 signaling on Porphyromonas gingivalis LPS-induced cardiac dysfunction in mice
Source: PLoS One. 2022 Jun 1;17(6):e0258823. doi: 10.1371/journal.pone.0258823 (PMC9159598; doi:10.1371/journal.pone.0258823)
Supplement: Attachment — Submitted filename: Review Plos one.docx [file pone.0258823.s003.docx]

Thank you for submitting to PLOS ONE.

I’m very interested in your article “Role of TLR4 signaling on Porphyromonas gingivalis LPS-induced cardiac dysfunction in mice”.

I recognize that the main points of this paper are the following two.

Immune system activation induced by Lipopolysaccharide on gram-negative bacterial pathogen known to be the cause of periodontitis, induce cardiac dysfunction and myocardial remodeling via the TLR4 signal. Furthermore, it was suggested that the TLR4-NOX4 pathway previously reported in the heart failure model may play an important role.

I sound that this paper is suitable for publication in PLOS ONE

However, one major revisions and some minor revisions are required.

Major revisions：

The target of your study is myocardial cell damage and cardiac dysfunction. However, cardiovascular events mediated by arteriosclerosis are discussed in introduction. Originally, if you would consider cardiovascular events related to atherosclerosis, coronary artery or vessels must be also investigated. I think introduction should focus on myocardial cell damage. For example, how about changing the reference 1) 12) to other paper such as heart failure model related to myocardial cell damage and discussing it?

Minor revisions：

1) You should write P-value at every item in all tables, even if there is no significant difference.
2) The order and abbereviation of the items in the Table2 A&B have to be changed as follows,

Order

1 EF

2 LVEDV

3 ESV → LVESV

4 FS

5 LVIDd →LVDd

6 LVIDs →LVDs

7 HR

8 SV

9 CO

10 IVSDd

11 LVPWTd →PWTd

Moreover, except LVSTs＆LVPWTs, and change Abbereviation details below.

EF (%): ejection fraction

LVEDV (mL): left ventricular end-diastolic volume

LVESV (mL): left ventricular end-systolic volume

FS (%): fractional shortening

LVDd (mm): left ventricular dimension at end-diastole

LVDs (mm): left ventricular dimension at end-systole

HR：heart rate

SV (mL): strole volume

CO (mL/min): cardiac output

IVSTd (mm): interventricular septum thickness at end-diastole

PWTd (mm): posterior wall thickness at end-diastole

3) The purpose of this study you wrote is twofold. On the other hand, only one is shown in the conclusion. Make sure that the purpose and the conclusion are matched as there is no discrepancy.
